# Freezing of Gait in Parkinson's Disease: Risk Factors, Their Interactions, and Associated Nonmotor Symptoms

**DOI:** 10.1155/2021/8857204

**Published:** 2021-01-12

**Authors:** David Gordon Lichter, Ralph Holmes Boring Benedict, Linda Ann Hershey

**Affiliations:** ^1^Department of Neurology, Jacobs School of Medicine and Biomedical Sciences, University at Buffalo, Buffalo, NY, USA; ^2^VA Western NY Healthcare System, Buffalo, NY, USA; ^3^Department of Neurology, University of Oklahoma Health Sciences Center, Oklahoma City, OK, USA

## Abstract

**Background:**

Freezing of gait (FOG) is a debilitating and incompletely understood symptom in Parkinson's disease (PD).

**Objective:**

To determine the principal clinical factors predisposing to FOG in PD, their interactions, and associated nonmotor symptoms.

**Methods:**

164 PD subjects were assessed in a cross-sectional retrospective study, using the MDS-UPDRS scale, MMSE, and Clinical Dementia Rating Scale. Clinical factors associated with FOG were determined using univariate analysis and nominal logistic regression. Receiver operating characteristic curves were computed, to obtain measures of sensitivity and specificity of predictors of FOG. Subgroups of patients with FOG were compared with those without FOG, based on defining aspects of their clinical phenotype.

**Results:**

Relative to non-FOG patients, those with FOG had a longer disease duration, higher PIGD and balance-gait score, higher LED, and more motor complications (*p* < 0.0001) and were more likely to exhibit urinary dysfunction (*p* < 0.0003), cognitive impairment, hallucinations, and psychosis (*p*=0.003). The balance-gait score and motor complications, at their optimum cutoff values, together predicted FOG with 86% accuracy. Interactions were noted between cognitive dysfunction and both the Bal-Gait score and motor complication status, cognitive impairment or dementia increasing the likelihood of FOG in subjects without motor complications (*p*=0.0009), but not in those with motor complications.

**Conclusions:**

Both disease and treatment-related factors, notably LED, influence the risk of FOG in PD, with a selective influence of cognitive dysfunction in patients with balance-gait disorder but not in those with motor fluctuations. These findings may help to inform clinical management and highlight distinct subgroups of patients with PD-FOG, which are likely to differ in their network pathophysiology.

## 1. Introduction

Freezing of gait (FOG), defined as a “brief, episodic absence or marked reduction of forward progression of the feet despite the intention to walk,” [1] is a debilitating motor symptom in Parkinson's disease (PD), increasing the risk of falls and loss of independence. Although its pathophysiology remains incompletely understood, it is thought to reflect dysfunction in an integrated network of brain regions involved in locomotion [2, 3]. Previously identified factors predisposing to FOG in PD include longer disease duration [4–7], more severe motor disability [6–8], higher nontremor score or PIGD phenotype [4, 5, 8, 9], higher levodopa dose [7, 8, 10], motor fluctuations [6–8], hallucinations [8, 11, 12], and cognitive dysfunction, especially executive impairment [10–14]. The relative importance of these various factors in the genesis of FOG in PD and the ways in which such factors might interact has only recently started to receive attention [15]. In this study, we sought to better understand the predominant clinical factors predisposing to FOG in PD, their possible interactions, and associated nonmotor symptoms. In particular, we sought to clarify the role of cognitive dysfunction in PD-FOG and whether its influence is exerted independently or via an interaction with other risk factors.

## 2. Subjects and Methods

### 2.1. Study Design and Subject Selection

A retrospective, cross-sectional study was performed to clarify the primary clinical factors uniquely predisposing to FOG in PD, their interactions, and associated nonmotor symptoms. This study used the same study cohort and shares some of the methods of Lichter et al., and the methods description partly reproduces their wording [16].

The study subjects consisted of 164 patients with PD, as defined by the UK Brain Bank criteria [17], who were assessed at two tertiary movement disorder centers in Buffalo, NY. Two hundred and eighteen charts were reviewed, of which 54 patients were excluded from the final analysis: 37 had coexisting neurological, psychiatric, or medical conditions sufficient to contribute to impairments in balance, gait, or cognition; seven had an alternative or additional cause for dementia or parkinsonism; and two had received DBS surgery. In addition, eight patients were excluded based on missing elements of the clinical ratings or demographic data. For the 54 patients excluded, demographic data, total motor MDS-UPDRS score, and the MDS-UPDRS Pt 1 (total nonmotor experiences of daily living) score did not differ significantly from that of the patients included in the analysis (data not shown). Case inclusion required a minimum data set that included: diagnosis of idiopathic PD; at least one complete MDS-UPDRS rating [18], performed in an “on” state; and MMSE and Clinical Dementia Rating (CDR) [19], both recorded within 3 months of the MDS-UPDRS rating.

### 2.2. Demographic and Motor Data

Age of onset of PD was defined as the age at the first motor symptom of PD and disease duration as the period between the first motor symptom of PD and the MDS-UPDRS rating. Both motor and nonmotor symptoms of PD were evaluated by the MDS-UPDRS scale [18]. Motor subscores included assessments of bradykinesia, tremor, rigidity, the PIGD score (sum of scores for items 2.12, 2.13, 3.10, 3.11, and 3.12) [20], and the PIGD minus freezing or “Bal/Gait” score (the sum of scores for items 2.12, 3.10, and 3.12, i.e., a clinical rating concerning problems with walking and balance over the past week and observation of gait and postural stability, excluding ratings of freezing of gait) [16]. The motor phenotype at the time of the evaluation was defined by the tremor/PIGD (postural instability-gait disorder) ratio, as follows: (1) tremor-predominant (ratio ≥ 1.15); (2) PIGD (ratio ≤ 0.90); or (3) indeterminate (ratio = 0.90–1.15) [20].

### 2.3. Assessment and Definition of Freezing of Gait

FOG was identified and scored by the MDS-UPDRS Part II (Motor aspects of Experiences of Daily Living (EDL)) item 2.13 rating and Part III (Motor Exam) item 3.11 rating. As for the other motor scales, severity of FOG for both Part II (Motor EDL) and Part III (Motor Exam) ranged from 0 (normal) to 4 (severe), each rating secured by an anchored clinical description. For the purpose of the study, FOG was recorded as being present if endorsed at any level of severity on either part of the rating scale. Thus, the minimal data necessary to classify a patient as positive for FOG was a rating of 1 on item 2.13: viz, an endorsement of the response option “I briefly freeze, but I can easily start walking again. I do not need help from someone else or a walking aid (cane or walker) because of freezing” in response to the question “Over the past week, on your usual day when walking, do you suddenly stop or freeze as if your feet are stuck to the floor?” The corresponding minimal rating (1: slight) of FOG on the motor exam was the observation “freezes on starting, turning, or walking through doorway with a single halt during any of these events but then continues smoothly without freezing during straight walking.” We also assessed motor complications, including the presence of motor complications, functional impact of motor fluctuations (item 4.4), total motor fluctuations score (items 4.3–4.6), and total motor complications score (items 4.1–4.6, including both dyskinesias and motor fluctuations).

### 2.4. Assessment of Nonmotor Symptoms

Nonmotor symptoms of PD were evaluated using the MDS-UPDRS nonmotor (nM) symptoms questionnaire. Part 1A is administered by the rater, incorporating pertinent information from the patient and caregivers, and addresses six complex behaviors: cognitive impairment, hallucinations and psychosis, depressed mood, anxious mood, apathy, and features of dopamine dysregulation syndrome. Part 1B, a component of the self-administered patient questionnaire, covers seven nonmotor symptoms: sleep problems, daytime sleepiness, pain and other sensations, urinary problems, constipation problems, light headedness on standing, and fatigue. Patients are asked to assess their average function over the previous week, without attempt to separate Parkinson's disease from other conditions. The patient was the primary data source, but, where necessary, for example for some patients with dementia, ratings were provided either by the caregiver or, in equal proportion, by the patient and caregiver.

### 2.5. Definition and Assessment of Cognitive Impairment

All participants were screened for cognitive impairment. For diagnosis of mild cognitive impairment (MCI), we used a modification of the Movement Disorder Society (MDS) task force guidelines for PD-MCI [21]. This was consistent with the DMS-V criteria for mild neurocognitive disorder [22, 23] and recognized established findings of discriminant validity of the MMSE as a screening and diagnostic instrument for MCI in PD [24] as well as the lack of meaningful differences in performance between the MMSE and the MoCA as a screening or diagnostic tool for PD-MCI [25, 26]. A subgroup of subjects (*n* = 19, 12%) met the task force specific Level II guidelines for PD-MCI using a comprehensive neuropsychological assessment. The remainder underwent an abbreviated assessment, consistent with Level I guidelines but using the MMSE as the global cognitive scale and applying the previously defined optimal screening cutoff score of 29/30 for the MMSE for PD-MCI [24, 26]. For all subjects, the diagnosis of MCI was supported by the Clinical Dementia Rating Scale (CDR score = 0.5) [16, 19].

Dementia was defined by DSM-V criteria for major neurocognitive disorders [22] and the Movement Disorders Task Force Level 1 criteria for diagnosis of PDD [27]. This includes PD development prior to the onset of dementia, MMSE score below 26, cognitive deficits sufficiently severe to impact daily living (caregiver interview or Pill Questionnaire), and impairment in more than one cognitive domain. The latter included impairment in at least two of the following tests: serial seven subtraction; clock drawing or lexical fluency; MMSE pentagons; and 3-word recall. Also required was exclusion of other primary explanations for cognitive impairment such as delirium, stroke, metabolic or endocrinologic abnormalities, head trauma, or adverse effects of medication.

Ethical approval for the study was obtained from the US Veterans Health Administration (Western NY) Institutional Review Board, with waiver of informed patient consent, considering the retrospective nature of the study. Permission for use of the MMSE for the study was granted by Psychological Assessment Resources, Inc.

### 2.6. Statistical Analysis

For subjects with and without FOG, demographic variables, medication use, including levodopa equivalent dose (LED), the motor phenotype [20], frequency of cognitive impairment or dementia, and both motor and nonmotor subscores of the MDS-UPDRS were compared, using logistic regression (likelihood ratio test) for continuous, normally distributed data, the Wilcoxon rank sum test for nonparametric or ordinal data, and the Chi-square test for categorical variables. Variables associated with FOG at *p* value < 0.05 were examined in an initial univariate logistic regression analysis. Odds ratios (ORs) and 95% confidence intervals (CIs) for univariate predictors were assessed. Variables with high univariate predictive accuracy (R^2^) were then entered in a full-model, multivariate logistic regression analysis, a single variable being entered at a time for variables demonstrating multicollinearity (*R* > 0.5) [28]. Of note, we entered the Bal-Gait scale score (*R*^2^ = 0.15) and not the more highly predictive PIGD score (*R*^2^ = 0.31) in this analysis, in view of the inclusion of FOG ratings in the assessment of the PIGD score and phenotype [20] (see Discussion). Collinear variables with high univariate predictive accuracy were assessed in separate multiple logistic regressions. Variables with *P* values for effect likelihood ratio tests <0.05 in the logistic regressions were retained. A stepwise approach was used, comparing the receiver operating characteristic (ROC) curve for the best predictor with that including the next best indicator. For each regression, nonsignificance of the Hosmer–Lemeshow test was confirmed, to ensure goodness-of-fit of the model. Linear discriminant analysis was used to compute the ROC curves. The optimal cutoff values for each predictor of FOG were defined by Youden's index [29]. Measures of sensitivity, specificity, accuracy, and ORs for these predictors were calculated. Clinical differences between subgroups of patients with FOG, derived from the logistic regressions, were explored. For group comparisons, a conservative criterion of *p* < 0.005 was chosen for statistical significance, to lessen the likelihood of type 1 error caused by multiple comparisons. JMP7 software (SAS Institute Inc., Cary, NC) was used for the statistical analyses.

## 3. Results

### 3.1. Frequency and Severity of Freezing of Gait

Seventy of the 164 PD patients (43%) had experienced symptoms of freezing of gait (FOG) during the week prior to their evaluation, which was slight in 27 (16.5%) patients, mild in 22 (13%), moderate in 14 (8.5%), and severe in 6 (3.6%) subjects. On examination, 36 (22%) patients exhibited signs of FOG, which was slight in 14 (8.5%), mild in 13 (8%), moderate in 17 (10.4%), and severe in 4 (2.4%) subjects.

### 3.2. Demographic, Motor, and Treatment Characteristics

The demographic and motor characteristics of participants with and without FOG are shown in Table 1. Patients with FOG tended to be older and had a longer duration of PD and levodopa therapy. The FOG patients were more likely to exhibit the PIGD subtype of PD, both the PIGD and Bal-Gait Score (MDS-UPDRS) being highly correlated with disease duration (*r* = 0.48 and *r* = 0.41, respectively, *p* < 0.0001). Other motor symptoms of parkinsonism were also more severe in patients with FOG, including bradykinesia (*p*=0.0002). In addition, patients with FOG had more severe motor complications, including both motor fluctuations and dyskinesias (*p* < 0.0001). Participants with FOG also had a higher levodopa equivalent dose (LED) (*p* < 0.0001) and were more likely to be receiving amantadine. Amantadine was primarily used in patients with motor complications, specifically those with dyskinesias (24%, versus 4% in those without dyskinesias: *χ*^2^ = 13.09, *p*=0.0003). The frequency of amantadine use in subjects with both motor complications and FOG was comparable to that in subjects experiencing motor complications without FOG (24% versus 9%, *p*=0.13).

### 3.3. Frequency and Severity of Cognitive Impairment and Other Nonmotor Symptoms

In our study, 32 PD patients (19.5%) met criteria for dementia and 66 (40%) met criteria for MCI, while 103 patients (62.8%) were assessed as cognitively impaired by the MDS-UPDRS rating. The frequency of MCI was similar in patients with and without FOG (44% versus 36%, respectively; *p*=0.29), but there was an increased frequency of both dementia (*p*=0.009) and MCI or dementia (*p*=0.001) in those with FOG (Table 1).

Participants with FOG also exhibited a greater severity and frequency of other nonmotor symptoms (Table 2). In particular, in addition to cognitive impairment, urinary problems, lightheadedness on standing, and hallucinations and psychosis, as assessed by the MDS-UPDRS rating, were more frequent in patients with FOG. Neither frequency nor severity of anxiety differentiated FOG patients from non-FOG patients.

### 3.4. Subgroup Analyses, including Associations between Motor Complications and Levodopa Dose

In PD patients with the PIGD phenotype (*n* = 100), those with FOG (*n* = 57, comprising 89% of all study subjects with FOG) were more likely to have a longer PD duration (*p*=0.0003), more severe motor symptoms, including higher balance-gait score (*p*=0.0009), a higher LED (*p*=0.002), and more severe motor complications (*p*=0.003) relative to those without FOG. They also showed a higher total nonmotor EDL score (*p*=0.0005), with a trend to more frequent urinary problems (74% versus 49%, *p*=0.01) and psychosis (25% versus 7%, *p*=0.03), but similar age and cognition to those without FOG.

In participants with motor complications (MC^+^, *n* = 73), those with FOG (*n* = 41, 64% of all subjects with FOG), relative to those without, were likely to be older (*p* < 0.02), with a longer duration of both PD and LD therapy (*p* < 0.0001), had a higher LED dose (*p* < 0.003) and more severe parkinsonism, including greater bradykinesia (*p*=0.007), and had a higher PIGD score (*p* < 0.0001) and balance-gait score (*p*=0.0007).

On further analysis, MC^+^ patients who had a LED ≥ 750 mg/day had more frequent FOG than those with a lower LED (67% versus 33%, *p*=0.009), but there was no relationship between LED and FOG severity, assessed by combined historical (Part II) and examination (Part III) FOG scores (LED = 1124 ± 320 mg versus 1089 ± 520 mg for patients with combined FOG score <2 (*n* = 112) versus ≥2 (*n* = 52), respectively, *p*=0.40). Conversely, in all patients with LED ≥ 750 mg, those with motor complications had a similar frequency of FOG relative to those without motor complications (67% versus 45%, *p*=0.09). Relative to those without FOG, MC^+^ patients with FOG showed a trend toward higher total nonmotor EDL score (*p* < 0.009), with more frequent lightheadedness on standing (*p*=0.004), but similar cognition, frequency and severity of anxiety, and other nonmotor symptoms. On the other hand, subjects with motor complications in general exhibited more frequent anxiety than those without motor complications (47% versus 20%, *p*=0.0003).

### 3.5. Independent Predictors of Freezing of Gait and Selective Effect of Cognitive Impairment

Table 3 shows the results of the univariate logistic regression analysis on FOG status. LED and the MDS-UPDRS motor complications score were highly correlated (*R* = 0.60, *p* < 0.0001) and, as collinear variables, were entered into separate multivariate logistic regressions. Both models were well fitted (AUC 0.83) and included the MDS-UPDRS Bal-Gait score, all three of these major variables exerting strong unique predictive effects for FOG (*p* < 0.0001) (Table 4). Cognitive impairment (MCI or dementia) was not a unique predictive factor in either model, but significant interactions were noted between cognitive impairment and both the Bal-Gait score and motor complication status (Table 4). Specifically, cognitive impairment or dementia increased the likelihood of FOG in subjects without motor complications (*p*=0.0009), but not in those with motor complications (Table 5).

### 3.6. Accuracy of Clinical Predictors of Freezing of Gait

Table 6 shows the diagnostic accuracy of singular and combined clinical predictors of FOG, based on their optimal cutoff scores. The MDS-UPDRS Bal-Gait score, motor complications score, and LED had individual predictive accuracies ranging from 70% to 73%. The combination of the Bal-Gait and motor complications cutoff scores, the measures with the highest sensitivities and specificities for prediction of FOG, respectively, produced a composite measure with a predictive accuracy for FOG of 86%. The combination of the three strongest clinical predictors did not further increase predictive accuracy for FOG (84%).

## 4. Discussion

Our study showed that FOG in PD was related to disease duration, PIGD phenotype or Bal-Gait score, LED, motor complications, cognitive impairment, and symptoms of psychosis. The most important independent predictors of current FOG, excluding the PIGD phenotype which itself contains an assessment of FOG, were the Bal-Gait score (≥4: OR = 8.0), LED (≥750 mg: OR = 5.4), and motor complications score (≥3: OR = 4.8) (Table 6). Cognitive impairment interacted with both the balance-gait score and motor complications status to modulate FOG risk.

We found FOG in PD to be unrelated to sex and only weakly associated with patient age, consistent with the majority of other prospective studies [4, 8, 10–12, 30]. On the other hand, supporting extensive literature, we found disease duration to be strongly associated with FOG [4–7, 9, 12, 13, 31]. One previous prospective study failed to show an association between disease duration and incident FOG [8]. However, this study assessed the influence of disease duration per 10-year increase, which may be too crude a measure to capture such an association.

In our study, disease duration was correlated strongly with PIGD severity (*r* = 0.48, <0.0001) and with the MDS-UPDRS Bal-Gait score (*r* = 0.41, *p* < 0.0001), a measure of balance-gait impairment without consideration of freezing [16]. Significant impairment of gait and balance tends to develop later in the course of PD, a manifestation of more severe disease that may emerge later from other motor phenotypes [32], although some patients display the PIGD phenotype from disease onset [33]. There is a robust literature supporting the association of PIGD with FOG in PD [4, 8, 9, 15, 31, 34]. Reactive and dynamic postural control have been identified as the most affected postural control systems in FOG [34, 35], specifically weight-shifting impairments and inadequate scaling and timing of postural responses, especially when occurring under time constraints [36]. This appears to be related to loss of presynaptic inhibition for step initiation [37]. Other than balance-gait impairment, bradykinesia was also strongly correlated with FOG in our study, most other studies similarly supporting the relationship of more severe motor disability with an increased risk of FOG [4, 5, 9, 11, 13, 14].

Independent of balance-gait impairment, the presence of FOG in our study was associated with severity of motor complications (OR = 4.8). The increased risk of FOG in PD patients with motor fluctuations, including the relationship between “off” period FOG and its alleviation by dopaminergic replacement therapy, is well established [4–7, 13, 38]. Our study did not assess “off” versus “on” period freezing. However, our finding of a relationship between presence of FOG and the functional impact of motor fluctuations indicates that at least part of the relationship of FOG with motor complications (Table 1) is likely to relate to increased motor dysfunction during “off” periods.

Levodopa equivalent dose was strongly correlated with motor complications (*p* < 0.0001), supporting previous studies showing a strong relationship between levodopa dose and development of both wearing-off symptoms and dyskinesias [39], and was also confirmed as a significant independent risk factor for FOG in our logistic regression analysis. Levodopa dose has previously been linked to FOG in PD [7, 8, 10] and, in at least one study, was also shown to exert an effect on FOG independent from that of motor complications [8]. In our study, participants with motor complications who had a higher LED (≥750 mg) had more frequent freezing (67% versus 33%, *p*=0.009) but similar severity of freezing relative to those receiving a lower LED. On the other hand, patients with a LED ≥750 mg/day with motor complications had no more frequent FOG than those without such complications (67% versus 45%, *χ*2 = 2.85, *p*=0.09). This suggests the possibility of a somewhat greater independent role of LED compared with motor fluctuations in the genesis of FOG in PD. However, additional studies of PD patients stratified for “on” versus “off” period freezing will be required to clarify this further. Overall, our analyses suggest an additive or possibly synergistic effect between either LED or motor complications, on the one hand, and balance-gait disorder, on the other, in the predisposition to FOG in PD (Table 6).

As recently discussed [40], it is difficult to reconcile levodopa's beneficial motor effects with its unique association with FOG. Indeed, some aspects of FOG, including slowed execution of the anticipatory postural adjustments for self-generated stepping, FOG frequency and duration, but not gait asymmetry and arrhythmicity, may improve in response to dopaminergic medication [38, 41]. In addition, in more advanced patients and those with motor fluctuations, a total of eight studies, including both retrospective and prospective open-label studies, have shown that levodopa-carbidopa intestinal gel improved FOG significantly [42–49]. Nevertheless, some forms of FOG, specifically freezing with attempted but ineffective stepping, has not been observed in untreated parkinsonian patients [50–52]. Historical observations suggest that FOG was rare before patients were treated with levodopa and reveal that the likelihood of FOG and the FOG phenotype changed after oral levodopa introduction, with FOG with attempted stepping then occurring more often [51–55]. This suggests, paradoxically, that pulsatile levodopa treatment may contribute to FOG, a phenomenon that has been postulated to be mediated by an increasing mismatch between activated cognitive and limbic loops but understimulated motor loops [40].

The relationship of medications other than levodopa to FOG in PD has also been evaluated. In a large cross-sectional study of FOG, freezers were treated with MAOIs and dopamine agonists without levodopa less frequently than nonfreezers, although it was acknowledged that this might reflect different treatment strategies in patients with earlier compared with more advanced disease stages [6]. A 3-year prospective study has suggested that the early use of selegiline, dopamine receptor agonists, or amantadine may be negatively related to FOG [11]. In addition, one double-blind randomized controlled trial and one open-label study showed a benefit of rasagiline as add-on treatment for FOG [56, 57], IV amantadine showing no benefit in two double-blind, randomized, placebo-controlled studies [58, 59]. FOG was also not improved by the acetylcholinesterase inhibitor rivastigmine in one double-blind placebo-controlled study [60]. In our cross-sectional study, no relationship was noted between prevalence of FOG and use of a MAO-B inhibitor, dopamine agonist, acetylcholinesterase inhibitor, SSRI, or SNRI. Amantadine was utilized almost exclusively for dyskinesias in our study patients experiencing motor fluctuations, and although its use was more common in subjects with FOG, the frequency of amantadine use in subjects with motor complications did not differ between subjects with and without FOG.

Although cognitive impairment was associated with FOG in the univariate analysis (OR = 3.0), the multivariate logistic regression showed that its influence was mediated via an interaction with balance-gait disorder, and only in the subgroup of patients without motor complications (Tables 4, 5). This adds context to the results of other studies that have shown FOG in PD to be associated with cognitive decline, particularly increasing visuospatial and executive dysfunction [30, 61, 62], including impairments in attention, response inhibition, and conflict resolution [63–67]. The contribution of cognitive processes to FOG is further supported by robust evidence that increased cognitive load may induce FOG [68]. Frontal cognitive functions are relevant to ambulation particularly when gait requires attentional resources, for example, when walking in unfamiliar environments [69], and executive dysfunction may contribute significantly to FOG during cognitively demanding tasks [70]. This is consistent with clinical observations that FOG in PD occurs most commonly in more challenging environmental conditions such as passage through narrow spaces, when turning, during a dual task, or while attempting to avoid obstacles. A resting-state functional MRI study has revealed disruption of connectivity of “executive-attention” and visual neural networks in PD subjects with FOG [71], and a voxel-based morphometry study has shown that PD patients with FOG can be distinguished by frontal and parietal atrophy, implicating brain regions involved in executive and perceptual functioning [72]. Other studies have also identified both structural and functional changes in frontal regions in PD patients with FOG [66, 72–79].

Unexpectedly, we found that the presence of cognitive dysfunction or dementia significantly increased the likelihood of FOG in PD patients without motor complications (*p* < 0.0009) but not in those with motor complications (Table 5). Compared with subjects with motor complications, PD patients without motor complications in our study had a later age of disease onset, shorter disease duration, and a significantly lower LED. This suggests a heterogeneous basis for FOG in PD in which cognitive dysfunction may aggravate FOG in some patients with a later age of disease onset through an interaction with abnormal motor circuitry, without similar effects in patients with earlier disease onset whose FOG may evolve more slowly over time in response to increasing levodopa dose and fluctuating levodopa availability. This hypothesis could be tested by evaluating the effect of cognitive load on FOG in PD patients with and without motor complications. In addition, a comparison of functional MRI patterns in PD-FOG patients with and without motor complications has the potential to shed light on possible differences in neural network dysfunction in these two groups.

In our study population, nonmotor symptoms differed somewhat between those patients whose FOG was associated with motor complications compared with those without motor complications who, broadly, displayed a prominent PIGD phenotype. As previously observed in PD patients displaying such a phenotype [16], subjects with PIGD-associated FOG lacking motor complications were characterized by cognitive impairment, dementia, and associated apathy, relative to those without FOG. In comparison, in those with motor complications, subjects with FOG demonstrated more orthostatic dizziness but similar cognition relative to patients without FOG.

PD subjects with FOG in our study exhibited not only increased cognitive dysfunction overall compared with those without FOG, but also a greater likelihood of hallucinations/psychosis (Table 2). The association of FOG with hallucinations/psychosis in PD has previously been reported [8, 11, 12, 30, 80–83]. We further showed that even in the subgroup of PIGD patients, those with FOG exhibited a trend to higher frequency of hallucinations/psychosis despite similar cognition relative to those without FOG.

The above findings suggest that the association of hallucinations and psychosis with FOG in PD is unlikely to be related simply to greater overall frontoparietal cortical dysfunction, as previously suggested [30], the relationship of psychosis with cognitive impairment and dementia in PD notwithstanding [16, 84–86]. It may be worth noting in this regard that PD patients with FOG exhibit abnormalities of both structural and functional connectivity and metabolism that overlap the findings observed in PD hallucinators. Just as evidence suggests dysfunction in posterior visual processing networks in PD hallucinators [87], both structural and functional neuroimaging studies have supported impairment of frontoparietal networks subserving visuospatial functions in PD-FOG [72–75, 79, 88–90], with white matter abnormalities and impaired connectivity in visual temporal areas [91]. The use of visual cues has been shown to alleviate FOG regardless of cognitive loading in dual task paradigms, providing further clinical support for the suggestion that altered sensory and particularly visual feedback may be an important factor in mechanisms of FOG in PD [92].

Finally, we noted that subjects with motor complications reported more frequent anxiety than those without motor complications, but, in subjects with motor complications, neither the frequency nor severity of anxiety was assessed as being greater in those with FOG compared with those without FOG. We did not, however, evaluate severity of anxiety using dedicated anxiety scales, the use of which has previously shown severity of anxiety to be higher in PD freezers compared with nonfreezers, except for those with mild FOG [93].

In summary, our findings indicate the importance of gait and balance disorder, LED, and motor complications, interacting with cognitive dysfunction, as the primary unique contributors to PD-FOG. This may be compared with the findings of Vercruysse et al. who found nongait freezing, LED, cognitive impairment, and falls and balance problems to independently predict PD-FOG [15]. The relative importance of LED as opposed to motor fluctuations, and specifically “off” time, as predictors of PD-FOG is not yet fully resolved and is confounded by the strong correlation between these variables. Nevertheless, our findings, together with clinical data from other studies, may help to inform the management of patients with PD, especially as “wearing-off” effects of levodopa develop.

There are some limitations of this study. Being retrospective in nature, the possibility of unintentional selection bias should be considered. However, the patients screened but not included in the study did not differ significantly in their demographic data or clinical ratings from the study patients. In addition, our study cohort presented a broad spectrum of disease severity and clinical profiles and appeared comparable to the general PD clinical population. The close screening of subjects to exclude other conditions that might affect balance, gait, or cognitive functioning strengthens the study. Use of the MoCA or other cognitive scales approved for the assessment of PD-MCI would allow greater generalizability of our findings, although it is likely that the great majority of our patients classified as MCI, who met current DSM-5 criteria for mild neurocognitive disorder, would also meet current consensus guidelines for PD-MCI [21, 94]. Our assessment of cognition was more limited than some previous studies but was sufficiently precise to allow a clear distinction between subjects with and without FOG and also to demonstrate the interaction between cognitive impairment and other major risk factors for FOG. Nevertheless, inclusion in future studies of more specific measures of executive and visuospatial functioning, typically impaired in PD and in PD-FOG, is likely to shed further light on pathophysiologic mechanisms underlying the influence of discrete cognitive processing deficits on PD-FOG, particularly in those subjects with nonfluctuating balance-gait disorder.

As typically employed in most studies of parkinsonian patients, we used a well-validated clinical measure to assess gait and balance, rather than posturography. We also employed both subjective and objective measures of FOG. While one previous study of FOG in PD included only patients with definite FOG, excluding those with self-reported freezing only [15], we considered that the clear description of FOG in the anchored MDS-UPDRS scale was sufficiently reliable to allow classification of patients with milder degrees of FOG purely on the basis of subjective symptoms. It is possible that use of the Freezing of Gait Questionnaire (FOG-Q) would have increased the sensitivity or accuracy of our subjective assessment [95]. We did not separate “on” from “off” or “on-off” freezing [6], limiting inferences that can be drawn from the association of FOG with motor complications.

Importantly, in this study, we dissected balance-gait impairment from the PIGD rating or phenotype [20], which include FOG measures, strengthening confidence in the linkage between non-FOG balance-gait disorder and FOG. As we were interested in risk factors for PD-FOG, which might provide insight into pathophysiologic mechanisms, we did not consider not-gait freezing, the inclusion of which, as a risk factor, in a previous PD study resulted in a model with a somewhat higher predictive strength for gait-freezing than our two models [15]. Our approach offers further insight into fundamental risk factors for PD-FOG and suggests avenues for future exploration of pathogenetic mechanisms and their interactions. Considering the cross-sectional nature of our study, however, further prospective studies of PD-FOG, somewhat limited to date [11, 96], will be important to better understand the evolution of factors contributing to PD-FOG and to evaluate strategies that might mitigate this risk at different stages of the disease.

## 5. Conclusions

Both disease and treatment-related factors, notably LED, influence the risk of FOG in PD, with an interacting influence of cognitive dysfunction in patients with balance-gait disorder, but not in the subgroup with motor fluctuations. These findings may help to inform clinical management and define PD populations, which may differ in their network pathophysiology. Further functional imaging studies in subgroups of FOG patients may increase understanding of the heterogeneous pathophysiology underlying PD-FOG, with the potential to advance management strategies in targeted subgroups.

## Figures and Tables

**Table 1:** Subject characteristics in relation to freezing of gait.

Variable demographics	FOG^+^	FOG^−^	*p* value
*n*	64	100	
Male (*n*, %)	45 (70%)	67 (67%)	0.66
Age (y, SD)	74.0 (10.0)	70.8 (9.7)	0.02
Age at PD onset (y, SD)	61.8 (12.1)	63.9 (11.0)	0.86
PD duration (y, SD)	12.1 (5.9)	6.9 (5.1)	<0.0001
Duration of L-Dopa Rx	8.7 (5.5)	3.8 (4.1)	<0.0001

Motor subtype (*n*, %)			
Tremor-predominant	5 (7%)	47 (47%)	<0.0001
Indeterminate	2 (3%)	10 (10%)	
PIGD	57 (89%)	43 (43%)	
Modified H/Y St. 1–2.5 (*n*, %)	28 (44%)	77 (77%)	<0.0001
Modified H/Y St. 3–5 (*n*, %)	36 (56%)	23 (23%)	
≥1 fall in past month (*n*, %)	36 (56%)	19 (19%)	<0.0001

MDS-UPDRS Subscale scores			
Pt. I: Nonmotor EDL	10.2 (4.5)	6.5 (4.0)	<0.0001
Nonmotor EDL score >7	47 (73%)	37 (37%)	<0.0001
Pt II: Motor EDL	19.4 (8.4)	10.8 (7.0)	<0.0001
Pt. III: Motor exam	39.5 (11.3)	31.3 (13.9)	<0.0001
Bradykinesia score	17.0 (4.9)	13.9 (5.9)	0.0002
Rigidity score	8.5 (3.1)	7.7 (3.7)	0.07
Tremor score	2.7 (4.1)	4.8 (4.6)	1.0
PIGD score	9.7 (4.8)	3.8 (3.1)	<0.0001
Balance-gait score	6.6 (3.3)	3.5 (2.9)	<0.0001
Pt. IV: Motor complications	3.8 (3.9)	1.2 (2.4)	<0.0001
Dyskinesias > 0 (*n*, %)	32 (50%)	18 (18%)	<0.0001
Time in off state > 0 (*n*, %)	32 (50%)	24 (24%)	0.0006
Functional impact of motor	—	—	—
Fluctuations > 0 (*n*, %)	31 (48%)	18 (18%)	<0.0001
Complexity of Mot. Fluct > 0	32 (50%)	22 (22%)	0.0002
Sum of Mot. Fluct. scores	2.5 (2.9)	0.9 (1.8)	<0.0001

Cognition			
Dementia (*n*, %)	19 (59%)	13 (13%)	0.009
Mild cognitive impairment or dementia (*n*, %)	48 (75%)	50 (50%)	0.001
Medications			
LED (mg, SD)	973 (493)	575 (391)	<0.0001
LD doses/day (mean, SD)	4.8 (2.0)	3.1 (1.9)	<0.0001
Dopamine agonist (*n*, %)	30 (47%)	35 (35%)	0.13
MAO-B inhibitor (*n*, %)	28 (44%)	46 (46%)	0.85
Amantadine (*n*, %)	12 (19%)	5 (5%)	0.005
Acetylcholinesterase inhibitor (*n*, %)	11 (18%)	9 (9%)	0.10
SSRI/SNRI (*n*, %)	16 (25%)	16 (16%)	0.18

FOG^+^ and FOG^−^ reference patients with and without freezing of gait, respectively. H/Y St: Hoehn and Yahr Stage; EDL: experiences of Daily Living; Mot. Fluct: motor fluctuations; LED: levodopa equivalent dose; LD: levodopa; MDS-UPDRS subscale scores are listed as mean (SD) and group scores as *n* (%).

**Table 2:** Frequency of nonmotor experiences of daily living in subjects with and without freezing of gait.

Nonmotor EDL	FOG^+^ (*n*, %)	FOG^−^ (*n*, %)	*p* value
Cognitive impairment	49 (77)	54 (54)	0.003
Hallucinations and psychosis	15 (23)	7 (7)	0.003
Depressed mood	19 (30)	28 (28)	0.82
Anxious mood	23 (36)	29 (29)	0.38
Apathy	16 (25)	11 (11)	0.02
Sleep problems	45 (70)	60 (60)	0.18
Daytime sleepiness	42 (66)	49 (49)	0.036
Pain and other sensations	18 (28)	25 (25)	0.66
Urinary problems	45 (70)	42 (42)	0.0003
Constipation problems	45 (70)	54 (54)	0.036
Light headedness on standing	21 (33)	12 (12)	0.001
Fatigue	32 (50)	51 (51)	0.90

**Table 3:** Clinical variables with high predictive accuracy for freezing of gait using univariate logistic regression analysis.

OR estimates					
Effect	Point estimate	95% confidence limits		*R* ^2^	*p* value
PIGD phenotype	10.79	4.48	26.00	0.18	<0.0001
MDS-UPDRS Bal-Gait score^1^	1.33	1.20	1.49	0.15	<0.0001
LED^2^	1.24	1.14	1.36	0.14	<0.0001
MDS-UPDRS motor complications score	1.30	1.16	1.47	0.11	<0.0001
Disease duration	1.18	1.11	1.26	0.14	<0.0001
Cognitive impairment^3^	3.00	1.51	5.97	0.05	0.001
Psychosis	4.07	1.55	10.64	0.04	0.003
Urinary problems	3.27	1.68	6.37	0.06	0.0003

ORs indicate the increase in odds for FOG resulting from a one-unit change in effect parameters: 1 rating point for an MDS-UPDRS rating scale score, or from 0 to 1 (effect absent to effect present), e.g., for cognitive impairment. For example, for a one-unit increase in the MDS-UPDRS Bal-Gait score, there is a 33% increase in the odds of developing freezing. ^1^The Balance-Gait score: the MDS-UPDRS PIGD score minus the freezing score. ^2^Levodopa equivalent dose (100 mg units). ^3^MCI or dementia.

**Table 4:** Results of multivariate nominal logistic regression on freezing of gait grouping.

Predictor	*β* estimate	Standard error	LR *χ*^2^^*∗*^	*p* value
*Model 1 (AUC* *=* *0.83, R*^2^ *=* *0.28)*				
MDS-UPDRS Bal-gait score^1^ ≥4	1.09	0.23	25.92	<0.0001
LED^2^	0.19	0.05	20.59	<0.0001
Cognitive impairment^3^	0.23	0.23	1.03	0.31
MDS-UPDRS Bal-Gait score ≥4 × cognitive impairment	−0.45	0.23	3.95	0.047

*Model 2 (AUC* *=* *0.83, R*^2^ *=* *0.29)*				
MDS-UPDRS Bal-Gait score^1^ ≥4	1.30	0.31	28.13	<0.0001
MDS-UPDRS motor complications score	0.41	0.12	21.75	<0.0001
Cognitive impairment^3^	0.22	0.25	0.82	0.36
MDS-UPDRS Bal-Gait score × cognitive impairment	−0.73	0.31	7.44	0.006
MDS-UPDRS motor complications score × cognitive impairment	−0.25	0.12	6.32	0.012

Variables with significant univariate predictive acuracy were entered in the multivariate logistic regression model. (Negative *β* estimations are the result of the class level design of the 1/−1 for 0/1 response variables in the SAS system). ^*∗*^Effect likelihood ratio chi-square test. ^1^MDS-UPDRS PIGD score minus freezing score. ^2^Levodopa equivalent dose (100 mg units). ^3^MCI or dementia.

**Table 5:** Association of cognitive status with freezing of gait in subjects with and without motor complications.

Motor complication status	Cognitive status^1^					
	Cognitive impairment	No cognitive impairment	*p* value	Dementia	No dementia	*p* value
	FOG (*n*, %)			FOG (*n*, %)		
Yes	29 (63%)	12 (44%)	0.12	9 (60%)	32 (55%)	0.74
No	20 (35%)	3 (9%)	0.0058	10 (59%)	13 (18%)	0.0009

^1^Cognitive impairment was defined as presence of MCI or dementia.

**Table 6:** Accuracy of clinical predictors of freezing of gait.

Clinical predictor	Sensitivity (%)	Specificity (%)	Accuracy (%)^1^	OR (95% CI)^2^
*Primary predictors*				
Bal-Gait score^3^ ≥ 4	80	67	72	8.0 (3.8–16.7)
LED^4^ ≥ 750 mg	69	71	73	5.4 (2.7–10.7)
Motor Compl. Score^5^ ≥ 3	55	80	70	4.8 (2.4–9.7)
MCI or dementia	75	50	60	3.0 (1.5–6.0)

*Combined predictors*				
Bal-Gait score ≥ 4 and LED ≥ 750 mg	84	79	81	20.2 (7.4–54.8)
Bal-Gait score ≥ 4 and motor Compl. score ≥3	87	86	86	40.4 (11.4–143.5)

^1^Accuracy, % of subjects correctly classified. ^2^Relative risk. ^3^Balance-Gait score = MDS-UPDRS PIGD score minus freezing score. ^4^Levodopa equivalent dose. ^5^MDS-UPDRS motor complications score.

## Data Availability

The data sets generated and analyzed during the current study are available at the US Department of Veterans Affairs Share Point repository (https://vaww.visn2.portal.va.gov/).
